# Supplementary material for: RodA promotes intestinal colonization by group B Streptococcus
Source: Infect Immun. 2026 Apr 30;94(6):e00730-25. doi: 10.1128/iai.00730-25 (PMC13248667; doi:10.1128/iai.00730-25)
Supplement: Supplemental Material — Figures S1-S6 and Table S1. [file iai.00730-25-s0001.pdf]

Supplementary Figures.

Supplementary Figure S1. Optimization of Lysozyme Concentration. Lysozyme concentrations were determined through preliminary testing of multiple concentrations (0 mg/ml, 12 mg/ml, 14 mg/ml and 20 mg/ml), with the final concentration of 14 mg/mL selected for the main experiment based on optimal cell lysis activity of 50% at 1 hour. Data from three technical replicates are shown, and lines represent mean values ± SDs.

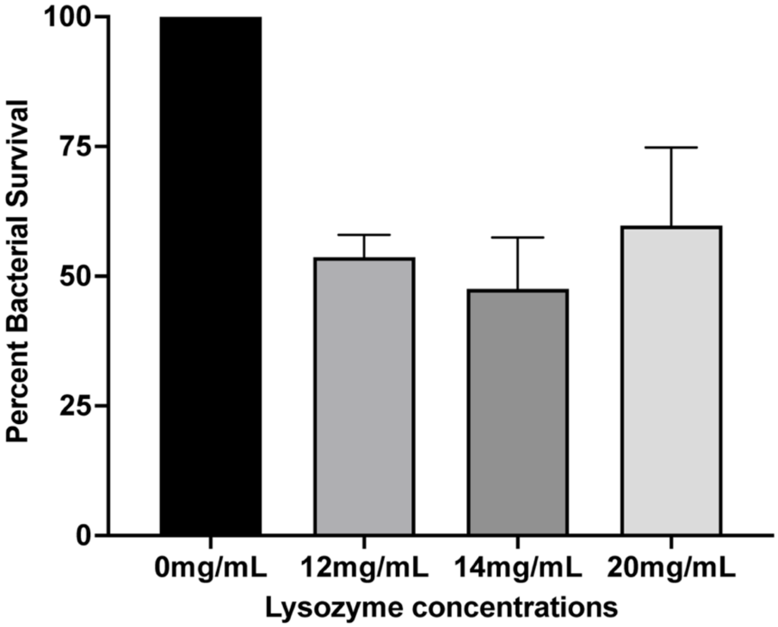

Supplementary Figure S2. A, B, C. Visualization of stationary-phase cultures grown for 18 hours at 37°C in Tryptic-Soy broth (TSB). **D, E.** The hemolytic activity of A909 WT and A909*ΔrodA* after 24-hour incubation on sheep blood agar.

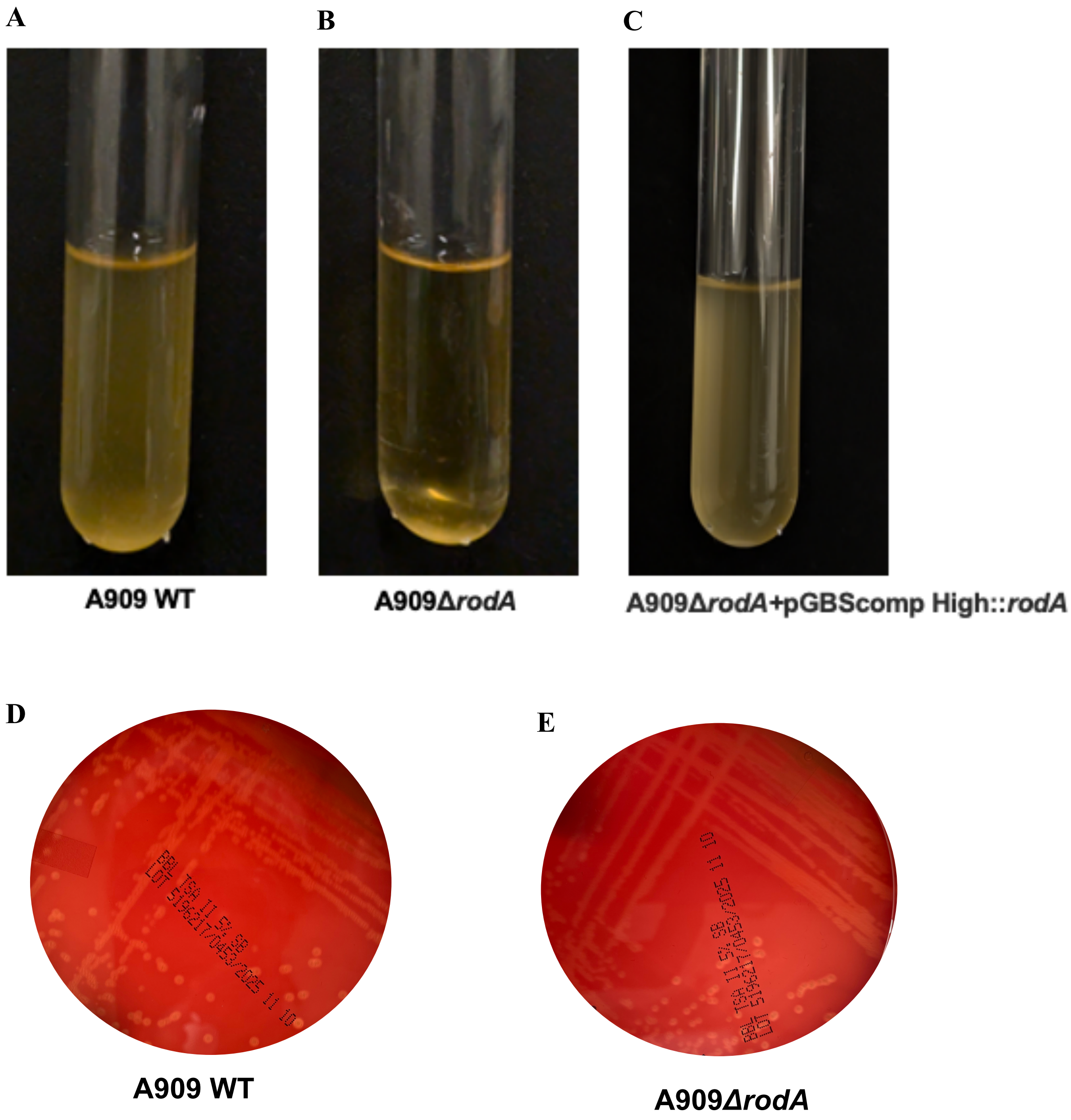

Supplementary Figure S3. Preweaning C57BL/6J mice were orally inoculated with a 1:1 mixture of A909 WT and A909Δ*rodA* in an experimental cohort (n = 5, 1 representative litter). **A.** Kaplan-Meier survival curve demonstrates 100% survival at day 7 post inoculation. **B.** Bacterial burden recovered across the GI tract at day 7 post inoculation.

A

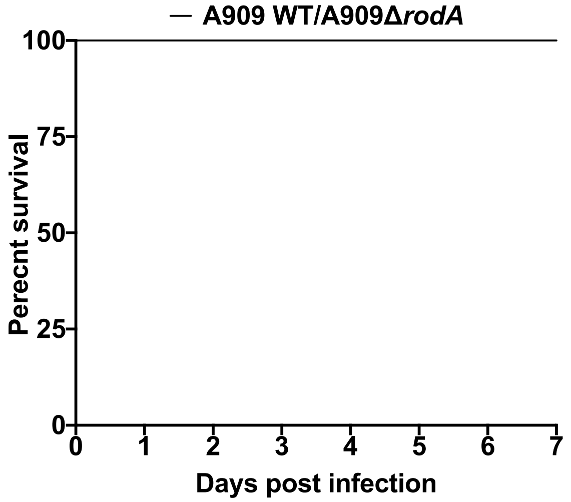

B

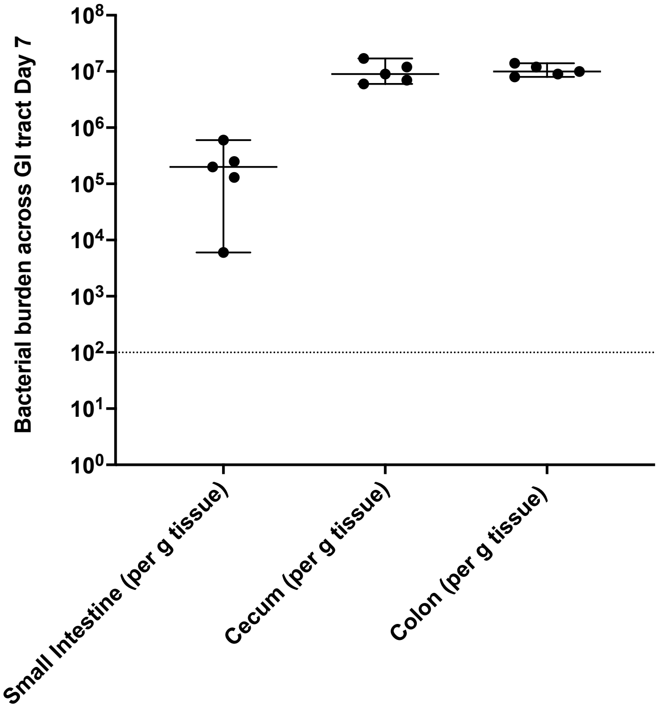

Supplementary Figure S4. Genotyping of bacterial colonies from the cocolonization experiment, recovered from the cecum. Representative 10 colonies were analyzed using cPCR, fractionated by electrophoresis in a 1% agarose gel. The left-most lane contains the 1kB DNA ladder, followed by A909 WT control (~3.3kB) and A909Δ*rodA* control (~2kB). The next nine lanes contain representative samples recovered from the cocolonization experiment.

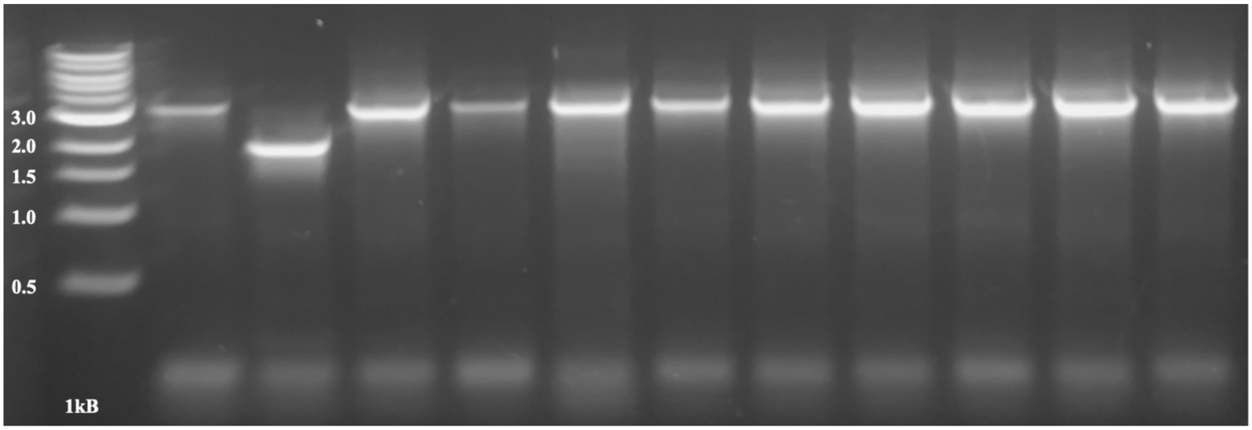

Supplementary Figure S5. Statistical analysis of growth kinetics under standard and stress conditions. **A, D**. Doubling time for A909 WT, A909Δ*rodA* and A909Δ*rodA*+pGBScomp High::*rodA* in TSB and Bile 5mg/ml, respectively. **B, E.** Area Under Curve (AUC) for A909 WT, A909Δ*rodA* and A909Δ*rodA*+pGBScomp High::*rodA* in TSB and Bile 5mg/ml, respectively. **C, F**. Maximum growth for A909 WT, A909Δ*rodA* and A909Δ*rodA*+pGBScomp High::*rodA* in TSB and Bile 5mg/ml, respectively. Scatter plot with medians indicated. Data were analyzed using Kruskal-Wallis test with Dunn's post-test for multiple comparisons (*, $p<0.05$; **, $p<0.005$; ***$p< 0.001$; ****$p< 0.0001$.

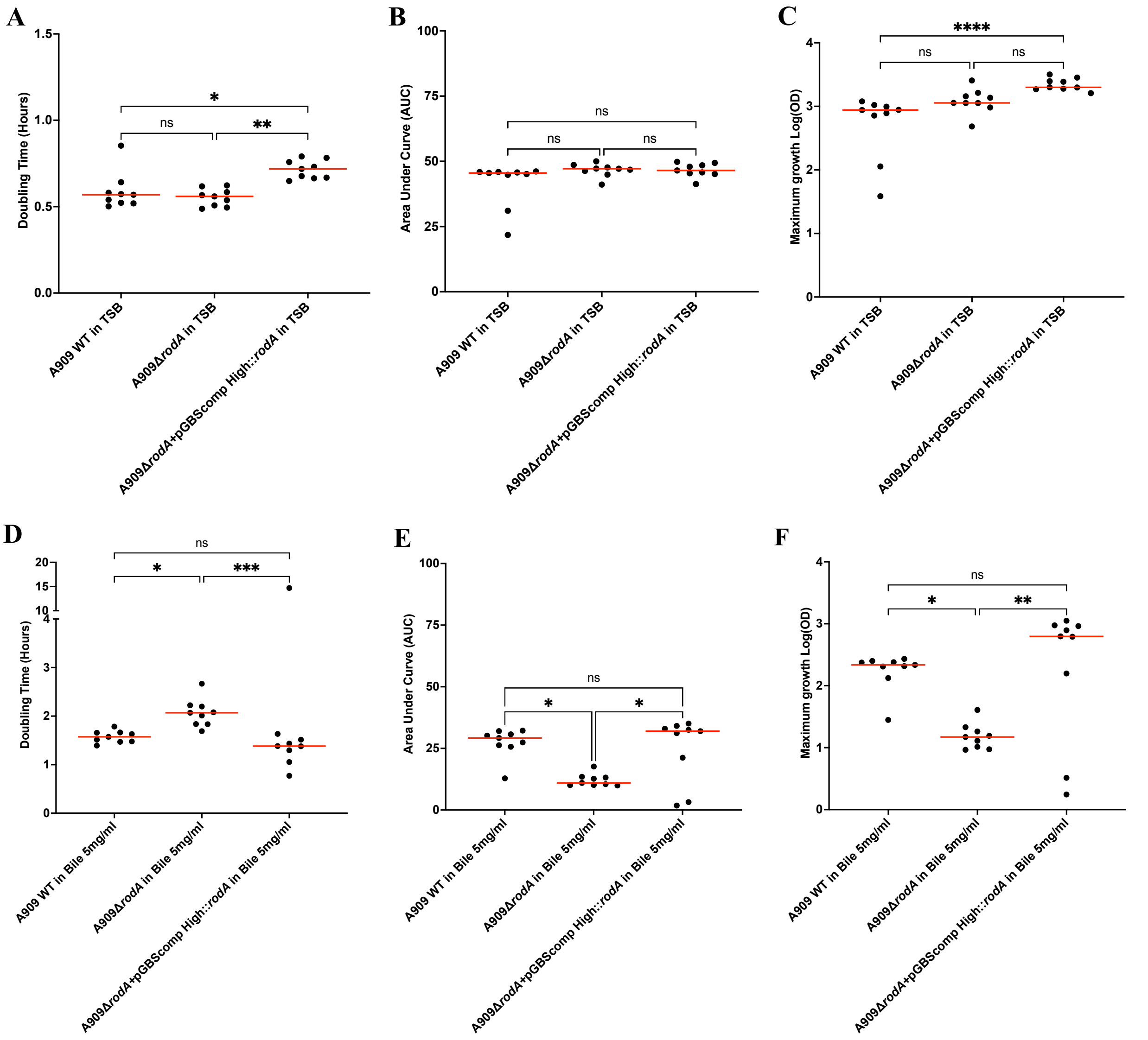

Supplementary Figure S6. Invasion efficiency of A909 WT and A909ΔrodA in intestinal epithelial cell lines. Invasion efficiency was calculated as invaded CFU divided by adherent CFU × 100 for each biological replicate. **A.** Caco-2 intestinal epithelial cell line **B.** T84 intestinal epithelial cell line. All data are from independent experiments (n=9, each with 3 technical replicates). The error bars indicate the 95% confidence intervals of the means of the wells. Data were analyzed using the Mann-Whitney U test *(****, $p < 0.0001$)*.

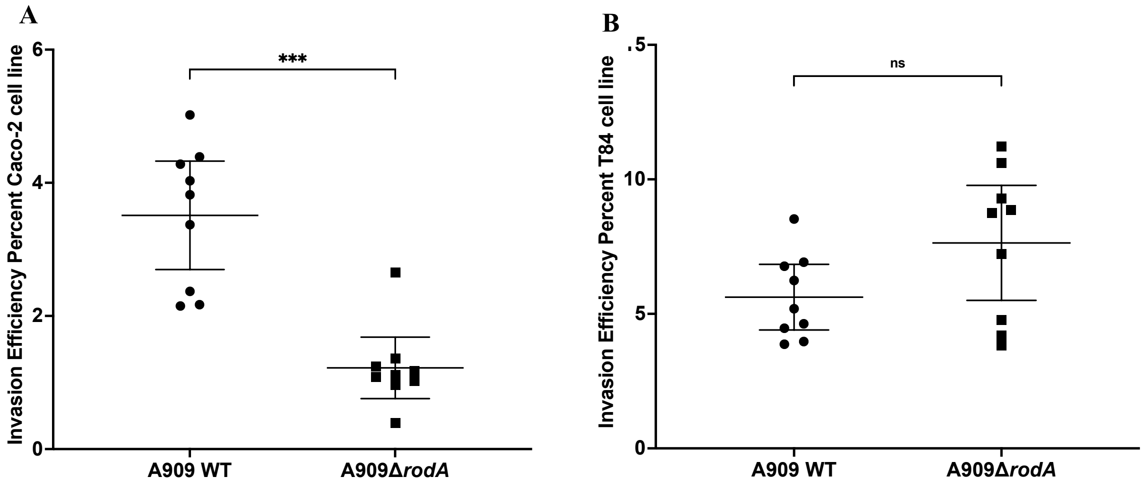

Supplementary Table 1. PCR Primers used in this study

Name	Sequence (5' to 3')	Notes
sgRNA	GGACTAACCCTAATGGTTCTTCC	crRNA sequence (functional RNA component that guides the enzyme to the target DNA).
RevCom sgRNA	GGAAGAACCATTAGGGTTAGTCC	Reverse complement to crRNA
Fwd Oligo for sgRNA	TAGAT-GGACTAACCCTAATGGTTCTTCC-A	Forward primer for crRNA
Rev Oligo for sgRNA	AAATT-GGAAGAACCATTAGGGTTAGTCC-A	Reverse primer for crRNA
rodA_Upstream_fwd	aatttctactcttgtagatcGAAGGCTTTGGCAAAATAAC	Homology arms: 780bP sequence upstream (forward) from the gene of interest.
rodA_Upstream_rev	cctttttttgAGGACTTCCTTTTTTATTCATG	Homology arms: 780bP sequence upstream (reverse) from the gene of interest.
rodA_Downstream_fwd	aggaagtcctCAAAAAAAGGTTGGAAAGATAATCCAGC	Homology arms: 780bP sequence downstream (forward) from the gene of interest.
rodA_Downstream_rev	agcttgcatgtctgcaggccCCTGGACGCATGCGAACAG	Homology arms: 780bP sequence downstream (reverse) from the end of the gene.
rodA_Chk_fwd	TAGCCATTTTAGGGAGCGCC	Chromosomal Screening Primers to screen and identify mutants: 750 bp upstream from the start gene.
rodA_Chk_rvs	TTACCACCGGCATGGAGAAC	Chromosomal Screening Primers to screen and identify mutants: 750 bp downstream of the end of the gene.
pGBSComp_rodA_fwd	tgacaatgatgttggatccg ATGTCACAAAAATCAAACTATTTTAAAG	Forward primer for complementary strain.
pGBSComp_rodA_rev	tcgataagcttggctgcagg TTATATACTACGATACTTCAAATGTG	Reverse primer for complementary strain.
